# Intestinal helminth infection transforms the CD4^+^ T cell composition of the skin

**DOI:** 10.1038/s41385-021-00473-9

**Published:** 2021-12-20

**Authors:** Cajsa H. Classon, Muzhen Li, Ada Lerma Clavero, Junjie Ma, Xiaogang Feng, Christopher A. Tibbitt, Julian M. Stark, Rebeca Cardoso, Emma Ringqvist, Louis Boon, Eduardo J. Villablanca, Antonio Gigliotti Rothfuchs, Liv Eidsmo, Jonathan M. Coquet, Susanne Nylén

**Affiliations:** 1 Department of Microbiology, Tumor and Cell Biology, Karolinska Institutet, 171 65 Solna, Sweden; 2 Development and Homeostasis of the Nervous System Laboratory, The Francis Crick Institute, London, NW1 1AT United Kingdom; 3 Department of Medicine, Solna, Karolinska Institutet and University Hospital, 17176 Stockholm, Sweden; 4 Center for Molecular Medicine, 17176 Stockholm, Sweden; 5 Department of Medicine, Huddinge, Karolinska University Hospital, 141 86 Stockholm, Sweden; 6 Polpharma Biologics, Utrecht, The Netherlands; 7 Department of Microbiology and Immunology, LEO Foundation Skin Immunology Research Center, University of Copenhagen, DK-2200 Copenhagen, Denmark

## Abstract

Intestinal helminth parasites can alter immune responses to vaccines, other infections, allergens and autoantigens, implying effects on host immune responses in distal barrier tissues. We herein show that the skin of C57BL/6 mice infected with the strictly intestinal nematode *Heligmosomoides polygyrus* contain higher numbers of CD4^+^ T cells compared to the skin of uninfected controls. Accumulated CD4^+^ T cells were *H. polygyrus-*specific T_H_2 cells that skewed the skin CD4^+^ T cell composition towards a higher T_H_2/T_H_1 ratio which persisted after worm expulsion. Accumulation of T_H_2 cells in the skin was associated with increased expression of the skin-homing chemokine receptors CCR4 and CCR10 on CD4^+^ T cells in the blood and mesenteric lymph nodes draining the infected intestine and was abolished by FTY720 treatment during infection, indicating gut-to-skin trafficking of cells. Remarkably, skin T_H_2 accumulation was associated with impaired capacity to initiate IFN-*γ* recall responses and develop skin-resident memory cells to mycobacterial antigens, both during infection and months after deworming therapy. In conclusion, we show that infection by a strictly intestinal helminth has long-term effects on immune cell composition and local immune responses to unrelated antigens in the skin, revealing a novel process for T cell colonisation and worm-mediated immunosuppression in this organ.

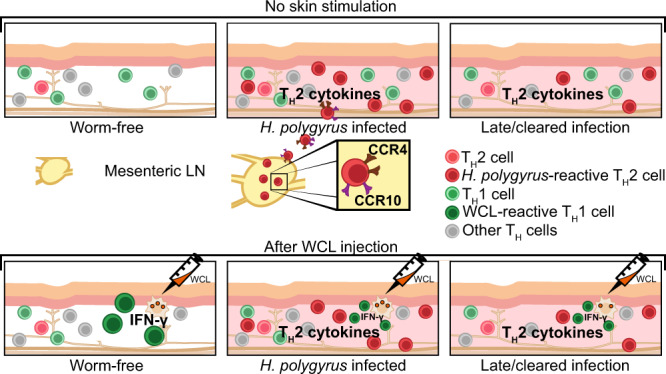

## Introduction

Immune responses in the gut and skin are deeply intertwined, as indicated by skin manifestations of intestinal disorders such as inflammatory bowel disease, Coeliac disease, small intestinal bacterial overgrowth and food allergies^1^. The reduced prevalence of helminth infections in modern times has been linked to an increase in autoimmune and inflammatory disorders in Western societies^2,3^. Intestinal worm infections have been suggested to have beneficial effects on inflammatory skin disorders with reduced atopy and allergic reactions in the skin^2,3^. We and others have shown that worm infection can dampen immune responses to infection and vaccination in the skin^4–6^. Enhanced T helper cell type 2 (T_H_2) and regulatory (T_REG_) responses are commonly suggested to drive the down-modulating effects of worms on subsequent immune responses^2^. Furthermore, we recently described how a chronic intestinal infection with the model worm pathogen *Heligmosomoides polygyrus bakerii* causes redistribution of lymphocytes with the accumulation of T cells in the mesenteric lymph nodes (mesLNs)^6^. Worm-induced expansion of mesLNs resulted in atrophy and loss of T cells in skin-draining lymph nodes (LNs), leading to dampened responses in the skin to the tuberculosis vaccine *Mycobacterium bovis* Bacillus Calmette–Guérin (BCG)^6^.

Tissue-resident T cells (T_RMs_) in barrier tissues contribute to infection- or vaccine-driven immunity against pathogens and also to inflammatory disorders^7–11^. Given the dampening effect of worms on skin inflammation and the importance of skin T_RMs_ in local immune responses, we here assessed the impact of intestinal infection with *H. polygyrus* on skin T cell composition and function*. H. polygyrus* infects orally through intake of L3 larvae and distributes strictly to the gut where it establishes a persistent, chronic infection. The responses to *H. polygyrus* in the local intestinal mucosa are well described^2,6,12^. Alike other intestinal worm infections, *H. polygyrus* induces a T_H_2 response causing goblet cell hyperplasia and increased peristaltic movements that provoke worm expulsion. In the chronic phase of infection, T_REG_ responses dominate and ameliorate the pathological immune responses^2,6,12^. As mice infected with *H. polygyrus* loose lymphocytes from skin-draining LNs^6^, we hypothesised that intestinal worms would also affect the lymphocyte composition in the skin. Surprisingly, *H. polygyrus* promoted the accumulation of worm-specific T_H_2 cells in the skin which remained for months after worm expulsion. Interestingly, skin in situ recall responses to mycobacterial lysate was reduced in mice with chronic *H. polygyrus* infection as well as those treated from the infection. We propose that skin-deposited T_H_2 cells contribute to the altered skin immune response upon subsequent pathogen challenge or intradermal delivery of vaccines, providing a novel process to previously observed immune regulations in the skin of worm-infected mice.

## Results

### Intestinal *H. polygyrus* infection dampens skin recall responses

**Fig. 1:**
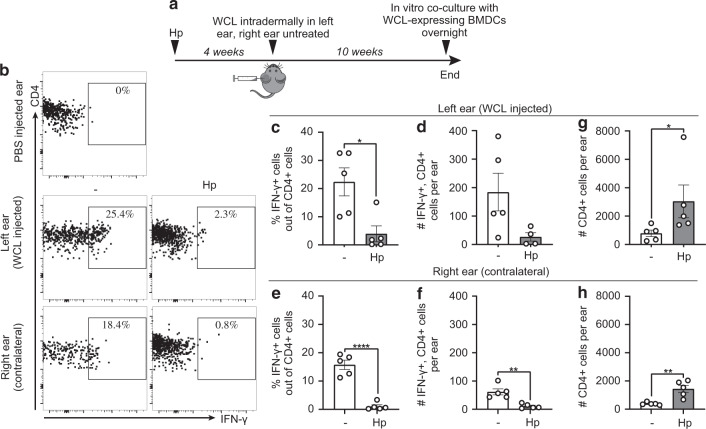
Intestinal *H. polygyrus* infection dampens recall responses to mycobacterial antigens in the skin. Mice were infected with *H. polygyrus* (Hp) or not (-) and treated according to the experimental outline **a** Four weeks after Hp infection, one ear was injected intradermally with whole-cell lysate (WCL) from *Mycobacterium tuberculosis*, and the contralateral ear was left untouched. Ten weeks later, ear cells were co-cultured with BMDCs expressing WCL overnight and analysed by flow cytometry. **b** Representative FACS plots illustrating the gating strategy used to identify WCL-specific IFN-*γ*^+^ CD4^+^ T cells. **c**–**f** Frequencies and absolute numbers of IFN-*γ*^+^ , CD4^+^ T cells in WCL injected and contralateral ears, respectively. **g**, **h** Absolute numbers of CD4^+^ T cells per ear in WCL injected and contralateral ears, respectively. One out of two independent experiments with similar results are shown. Each dot represents one mouse (*n* = 5 per group) and bars indicate the mean ± SEM. Statistical differences were analysed by Mann–Whitney U test (**c**, **g**) or unpaired *t*-tests (**d**–**f**, **h**) and depicted as *p < 0.05, **p < 0.01, and ****p < 0.0001.

We have previously shown that mice infected with *H. polygyrus* mount weaker delayed-type hypersensitivity (DTH) responses to BCG^6,13^. To investigate the effects of intestinal worms on the development of mycobacteria-specific memory T cells in the skin, local cytokine recall responses were assessed 5–10 weeks after injection of whole cells lysate (WCL) from *M. tuberculosis* in ear skin of worm-infected mice (Fig. 1a). WCL was used to avoid confounding factors associated with live mycobacterial challenges, such as microbial spread and replication. The long interval between WCL injection and recall assessment was used so that WCL-induced inflammation and effector responses would have waned. Ear thickness was measured at various time points after WCL administration, but no significant difference was observed between worm-infected and worm-free mice (Supplementary Fig. 1a). As ear thickness was not fully restored but rather stabilised with time, we also analysed the contralateral, untouched ear. Interestingly, WCL-specific recall IFN-*γ* production in both WCL-injected and untreated ears were lower in *H. polygyrus-*infected mice compared to worm-free controls (Fig. 1b–f, Supplementary Fig. 1b). WCL-specific cells were not pushed to produce T_H_2 cytokines (IL-4/IL-13) instead of IFN-*γ* (Supplementary Fig. 1c). IFN-*γ* production was dampened in worm-infected mice also in response to polyclonal restimulation but the numbers of Tbet^+^ CD4^+^ T cells (T_H_1) were not altered by the infection (Supplementary Fig. 1d–f). Instead, the median fluorescent intensity of IFN-*γ* in CD4^+^ T cells was reduced (Supplementary Fig. 1g), together indicating dampened functionality rather than reduced skin infiltration of the T_H_1 cells. Surprisingly, total CD4^+^ T cell numbers were higher in ears of *H. polygyrus-*infected mice in both WCL-treated and untreated ears (Fig. 1g, h). Hence, mice infected with *H. polygyrus* mount weaker skin recall responses to mycobacterial WCL both at the site of injection and at distal skin sites, despite an accumulation of CD4^+^ T cells in the skin.

### Intestinal *H. polygyrus* infection alone causes CD4^+^ T cells to accumulate in skin

**Fig. 2:**
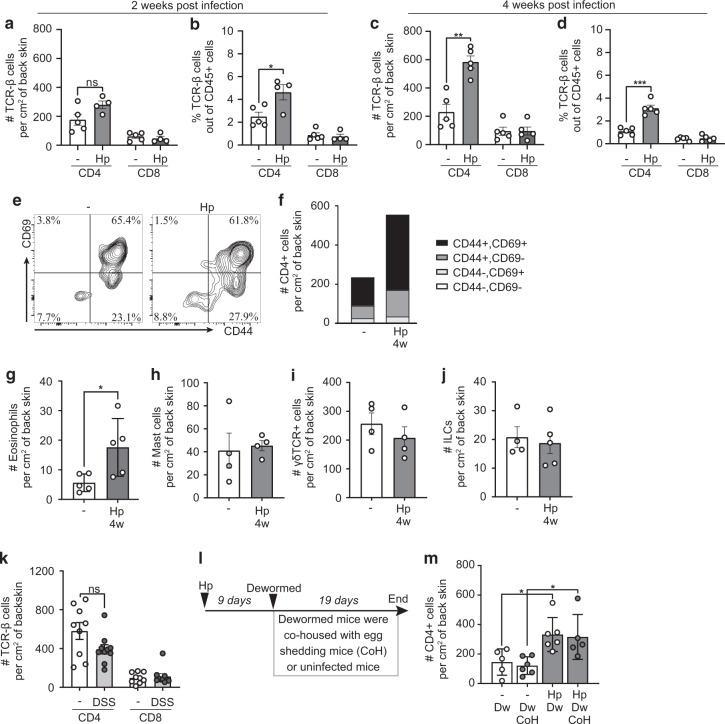
Intestinal *H. polygyrus* infection alone causes T cells to accumulate in skin. **a**–**j**, **m** Mice were infected with *H. polygyrus* (Hp) or not (−) and some dewormed (Dw) 4 weeks later. **a**–**d** Absolute numbers and frequencies (out of total CD45^+^ cells) of CD4^+^ and CD8^+^ T cells found in back skin 2 and 4 weeks post-infection. **e** Representative FACS plots of CD44 and CD69 gating of skin CD4^+^ T cells. **f** Absolute numbers and percentages of CD44 and CD69 out of CD4^+^ T cells. Data is calculated from an average of three independent experiments with a total of ≥12 mice per group. **g** Absolute numbers of eosinophils (CD11b^+^, Siglec-F^+^, CD45^+^), **h** mast cells (c-Kit^+^, FcεRI^+^, CD45^+ ^), **i** *γ*δ TCR^+^ (CD45^+^ ) cells and **j** ILCs (CD45^+^, lineage-, CD90.2^+^). **k** Mice were treated for 2 × 7 days with 2% dextran sodium sulphate (DSS) in the drinking water with 2 weeks in between the treatments and absolute numbers of CD4^+^ and CD8^+^ T cells in back skin is shown. **l** Experimental outline for **m** De-wormed mice were co-housed with either uninfected or infected (CoH), egg-shedding mice. Egg-shedding mice had been infected with 500 L3 Hp to ensure high egg production. **a**–**j**, **l**, **m** One out of at least two independent experiments with similar results are shown. In **j** the experiment has been performed once but with 10 mice per group (divided in two cages). Each dot represents one mouse (*n* ≥ 4 per group) and bars indicate the mean ± SEM. Statistical differences were analysed by unpaired *t*-tests (**a**–**d**, **g**–**k**) or one-way ANOVA with Tukey post-test (**m**), and depicted as ns = non-significant, *p < 0.05, **p < 0.01, ***p < 0.001.

We next analysed T cells in the skin of worm-infected mice without further manipulation. Succeeding experiments were performed on back skin, providing a larger surface area. Two weeks after *H. polygyrus* infection, skin CD4^+^ T cell numbers began to increase (Fig. 2a, b, Supplementary Fig. 2a) and by 4 weeks after infection T cell numbers were 2–3 times higher than in the skin of uninfected mice (Fig. 2c, d). No differences were noted in CD8^+^ cell numbers (Fig. 2a–d). Most CD4^+^ T cells in the skin of both worm-infected and worm-free mice co-expressed CD44 and CD69, indicating an effector or memory phenotype (Fig. 2e, f).

Mast cells and eosinophils are known to expand upon helminth infection^12,14,15^ and there were more eosinophils in the skin of *H. polygyrus*-infected mice (Fig. 2g, Supplementary Fig. 2b). Mast cells were clearly expanded in the spleen (Supplementary Fig. 2c–e), but no difference was found in the skin (Fig. 2h, Supplementary Fig. 2f, g). There were no differences noted in other myeloid cell populations such as neutrophils, macrophages, dendritic cells (DCs) or frequency of DCs expressing CD11b (pro-inflammatory DCs, Supplementary Fig. 2h–o). Innate lymphoid cells (ILCs) and *γ*δ T cells are common in barrier tissues in mice, but there was no difference in the skin of either cell type after worm infection (Fig. 2i, j, Supplementary Fig. 2p, q).

Mouse skin comes into contact with faeces, and intestinal helminth infection has been shown to increase intestinal permeability and bacterial translocation from the gut^2^. We next tested if T cell priming by intestinal bacteria in combination with environmental skin exposure to bacteria of faecal origin could drive T cell accumulation in the skin. Indeed, *H. polygyrus-*infected mice had elevated levels of soluble CD14 (sCD14) in serum (Supplementary Fig. 2r), an indirect marker for increased levels of LPS in blood^16^, suggesting enhanced bacterial translocation from the gut to the blood. To investigate if the increase in CD4^+^ cells in the skin was due to bacterial translocation, we measured CD4^+^ cell numbers in the skin of mice with colitis induced by dextran sodium sulphate (DSS) treatment, which is known to increase LPS levels in the blood^17^. DSS-induced colitis was established over a timeline similar to chronic *H. polygyrus* infection (Supplementary Fig. 2s) previously shown to initiate a T cell-driven inflammatory response in the intestine^18^. This protocol did not increase CD4^+^ T cell numbers in the skin (Fig. 2k). To further address the possibility that  altered skin infiltration would be driven by gut inflammation/infection per se, we infected T cell receptor (TCR) transgenic mice (p25-TCR Tg mice) with *H. polygyrus*. These mice have TCRs specific for an unrelated peptide (Ag85 peptide of mycobacteria)^19^, and are hence not capable of mounting a T cell response to the worms. As expected due to the lack of commensal-specific T cells, these mice had few T cells in the skin, and there was no increase in CD4^+^ T cell numbers after *H. polygyrus* infection despite a clear expansion of cell numbers in the mesLNs (Supplementary Fig. 2t, u). Together, this data indicates that bacterial translocation and/or generalised inflammation were not the cause of higher CD4^+^ T cell numbers in the skin.

To dismiss that worm product excreted in mouse faeces caused T cell recruitment to the skin, *H. polygyrus*-infected mice were dewormed early after infection when adult worms have left the intestinal wall but not started to produce eggs^12^. Cages were changed after deworming to minimise skin exposure to faecal antigens. Dewormed mice were then co-housed with heavily *H. polygyrus-*infected mice or uninfected controls (Fig. 2l). No difference in CD4^+^ T cell numbers was detected in skin collected from these groups (Fig. 2m; HpDw and HpDw CoH), but in line with previous experiments, CD4^+^ T cell numbers were increased in previously infected mice compared to uninfected controls irrespective of co-housing partners (Fig. 2m). This indicates that the gut infection rather than environmental worm antigen is responsible for CD4^+^ T cell accumulation in the skin.

### Skin-homing receptors are up-regulated on circulating CD4^+^ T cells after *H. polygyrus* infection

**Fig. 3:**
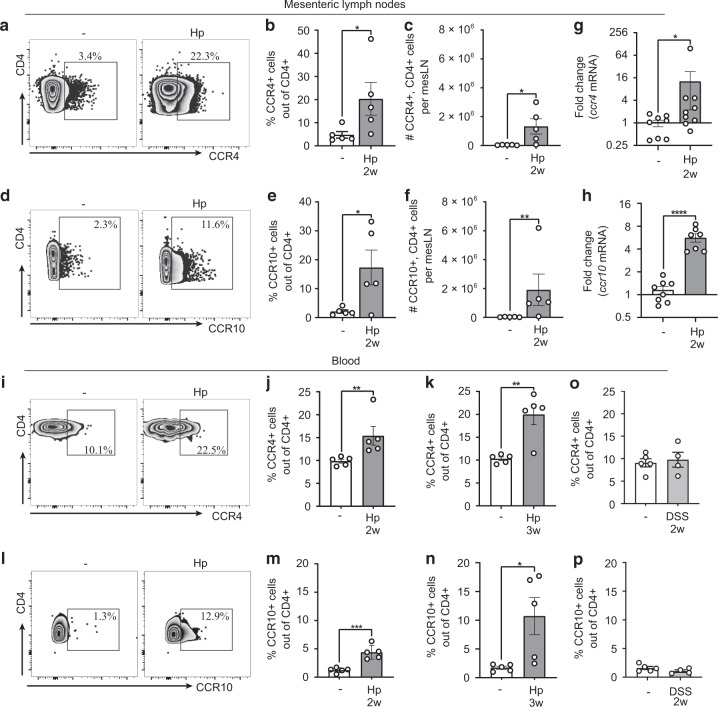
Skin-homing receptors are up-regulated on mesLN and circulating CD4^+^ T cells after *H. polygyrus* infection. **a**–**n** Mice were infected with *H. polygyrus* (Hp) or not (−). **a**–**h** Mesenteric lymph nodes (mesLNs) collected two weeks after infection. **a** Representative FACS plots of CCR4 staining gated on mesLN CD4^+^ T cells. **b**, **c** Frequencies and absolute numbers of CCR4^+^ cells out of CD4^+^ T cells. **d** Representative FACS plots of CCR10 staining gated on mesLN CD4^+^ T cells. **e**, **f** Frequencies and absolute numbers of CCR10^+^ cells out of CD4^+^ T cells. **g, h** Fold change of *Ccr4* and *Ccr10* mRNA expression in CD4^+^ T cells bead isolated from mesLNs. **i**–**p** Blood collected at indicated time points after infection. **i** Representative FACS plots of CCR4 staining gated on blood CD4^+^ T cells. **j**, **k** Frequencies of CCR4^+^ cells out of CD4^+^ T cells. **l** Representative FACS plots of CCR10 staining gated on blood CD4^+^ T cells. **m**, **n** Frequencies of CCR10^+^ cells out of CD4^+^ T cells. **o, p** Mice were treated for 7 days with 2% dextran sodium sulphate (DSS) in the drinking water and rested for 7 days. Frequencies of CCR4^+^ and CCR10^+^ cells out of CD4^+^ T cells, respectively. Samples were analysed by flow cytometry (**a**–**f**, **i**–**p**) or qPCR (**g**–**h**). In **a**–**f**, **i**–**p**, one out of at least two independent experiments with similar results are shown. **g**, **h** shows pooled data from two independent experiments. Each dot represents one mouse (*n* ≥ 4 per group) and bars indicate the mean ± SEM. Statistical differences were analysed by unpaired *t*-tests (**c**, **e**, **h**, **m**) or Mann–Whitney U test (**b**, **f**, **g**, **j**, **k**, **n**) and depicted as *p < 0.05, **p < 0.01, ***p < 0.001, ****p < 0.0001.

Tissue tropism is induced during the priming of T cells by the expression of homing receptors^20^. T cell trafficking to the skin depends on the expression of cutaneous lymphocyte-associated antigen (CLA) and the chemokine receptors (CCR) CCR4 and CCR10^20^. As expected, the mesLNs showed a great expansion of CD4^+^ cells after *H. polygyrus* infection (Supplementary Fig. 3a–c). Interestingly, expression of skin-homing receptors CCR4 and CCR10 was up-regulated in CD4^+^ cells from the mesLNs and in the circulation of worm-infected mice 2 and 3 weeks after infection (Fig. 3a–n, Supplementary Fig. 3d–f). As expected, expression of the gut-homing receptor CCR9^+^ ^20^ was also increased (Supplementary Fig. 3g–i). A 2-week DSS treatment regimen, however, did not up-regulate skin-homing receptors (Fig. 3o, p). This indicates that CD4^+^ T cells activated by *H. polygyrus* in mesLN are directed to the skin through up-regulation of skin-homing receptors.

### T_H_2 cells accumulate and persist in the skin after *H. polygyrus* infection

**Fig. 4:**
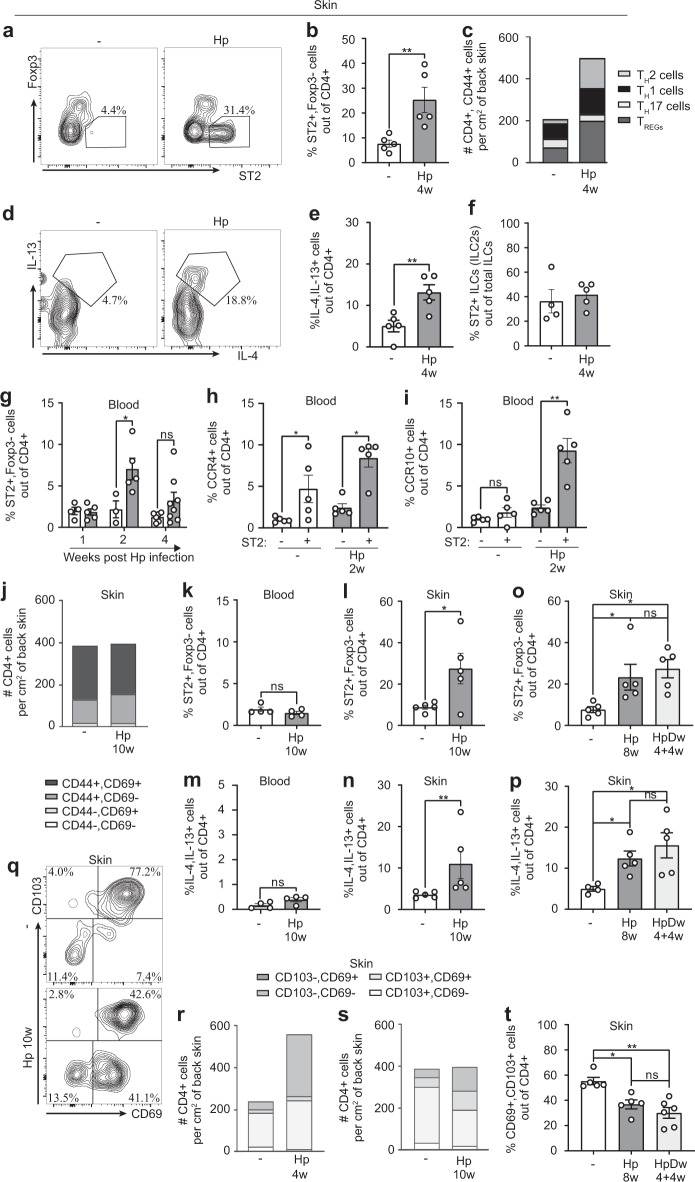
Type 2 CD4^+^ T cells accumulate and persist in skin after *H. polygyrus* infection. Mice were infected with *H. polygyrus* (Hp) or not (−) and some dewormed (Dw) 4 weeks later. **a**–**f** Flow cytometry of back skin cells. **a** Representative FACS plots of ST2^+^ , Foxp3^−^ staining gated on CD4^+^ T cells. **b** Frequency of ST2^+^ , Foxp3^−^ out of CD4^+^ T cells. **c** Absolute numbers and percentages of CD44^+^ T helper cell subsets (T_H_2 (ST2^+^ , Foxp3^−^), T_H_1 (Tbet^+^ , CXCR3^+^ ), T_H_17 (ROR*γ*t^+^ ) and T_REG_ cells (Foxp3^+^ ) out of CD4^+^ T cells. **d** Representative FACS plots of IL-13^+^ , IL-4^+^ cells gated on CD4^+^ T cells. **e** Frequency of IL-13^+^ , IL-4^+^ cells out of CD4^+^ T cells after PMA/ionomycin restimulation in the presence of Brefeldin A. **f** Frequency of ST2^+^ cells out of ILCs. **g**–**i** Flow cytometry of white blood cells. **g** Frequency of ST2^+^ , Foxp3^−^ cells out of CD4^+^ T cells. **h** Frequency of CCR4^+^ and **i** CCR10^+^ in the ST2 expressing (+) and non-expressing population (−) of CD4^+^ T cells. **j** Absolute numbers and percentages of CD44 and CD69 out of skin CD4^+^ T cells. Data is calculated from an average of three independent experiments with a total of ≥12 mice per group. **k**–**l**, **o** Frequencies of ST2^+^ , Foxp3^−^ cells out of CD4^+^ T cells in **k** blood and **l, o** skin. **m**, **n**, **p** Frequencies of IL-13^+^ , IL-4^+^ cells out of CD4^+^ T cells in **m** blood and **n, p** skin. **q** Representative FACS plots of CD103 and CD69 gating in skin CD4^+^ T cells. **r**, **s** Absolute numbers and percentages of CD103 and CD69 out of skin CD4^+^ T cells. Data is calculated from an average of three independent experiments with a total of ≥12 mice per group. **t** Frequency of CD103^+^ , CD69^+^ cells out of CD4^+^ T cells, mice were dewormed 4 weeks before analysis. One out of at least two independent experiments with similar results are shown. Each dot represents one mouse (*n* ≥ 4 per group) and bars indicate mean ± SEM. Statistical differences were analysed by unpaired *t*-tests (**b**, **e**, **h** (−), **i**, **l**), Mann–Whitney U test (**g**, **h** (**Hp**), **k**, **m**, **n**), Kruskal–Wallis with Dunn’s post-test (**o**, **t**) or one-way ANOVA with Tukey post-test (**p**) and depicted as ns = non-significant, *p < 0.05, **p < 0.01, ***p < 0.001.

Isolated studies have demonstrated that T_H_2 accumulate at extra-intestinal sites during worm infection, where they persist for at least 2 weeks after de-worming^21,22^. We next characterised the phenotype of the skin CD44^+^ , CD4^+^ T cell population after *H. polygyrus* infection. The frequencies and absolute numbers of T_H_2 cells (ST2^+^, Foxp3^−^ ^23^) were clearly increased in worm-infected mice (Fig. 4a–c, Supplementary Fig. 4a). Frequencies of other T_H_ cell subsets (T_H_1 (Tbet^+^ , CXCR3^+^), T_H_17 (ROR*γ*t^+^), T_REG_ cells (Foxp3^+^)) were either lower or unchanged, skewing the CD4^+^ T cell composition towards a T_H_2 profile (Fig. 4c, Supplementary Fig. 4b–i). Accordingly, skin of worm-infected mice contained a higher frequency of CD4^+^ cells that produced the T_H_2-associated cytokines IL-4 and IL-13 upon polyclonal re-stimulation (Fig. 4d, e). Whereas CD4^+^ T cells were clearly skewed towards a T_H_2 phenotype, there was no skewing of ILCs towards an ILC2 phenotype (Fig. 4f, Supplementary Fig. 4j). In the blood, T_H_2 cells were expanded 2 weeks after infection but had started to contract 2 weeks later (Fig. 4g). Interestingly, out of CD4^+^ blood T cells, T_H_2 (ST2^+^) cells expressed higher levels of CCR4 and CCR10 skin-homing chemokine receptors compared to ST2^−^ cells, a difference that was more pronounced in *H. polygyrus*-infected mice (Fig. 4h, i). Among ST2^+^ cells in infected mice, CCR4 was generally more strongly up-regulated than CCR9 and very few cells (<0.1%) were double-positive for the two markers (Supplementary Fig. 4k). T_H_2 cells seem, therefore, to be specially targeted to the skin in worm-infected mice.

To address if the worm-induced accumulation of CD4^+^ T cells in the skin persisted over time and following deworming treatment, we assessed skin CD4^+^ T cells for up to 10 weeks after infection, when most of our animals have naturally cleared the infection (Supplementary Fig. 4l). At this time point, the number of T cells had normalised in the skin and the frequency of activated cells (CD44^+^, CD69^+^) was still similar to that of uninfected and more recently infected mice (Fig. 2f, 4j). Although there were no signs of elevated circulating T_H_2 cells in long-term worm-infected mice, a higher frequency of T_H_2 cells persisted in the skin (Fig. 4k–n). Likewise, animals from which worms had been removed by drug treatment 4 weeks earlier retained as high frequencies of T_H_2 cells in the skin as animals still carrying the infection (Fig. 4o, p, Supplementary Fig. 4l).

Due to the longevity of T_H_2 cells in the skin, we next assessed the expression of memory markers on skin CD4^+^ T cells in our model. CD103 and CD69 are established T_RM_ markers for CD8^+^ T cells but no reliant markers have yet been established for tissue residency of CD4^+^ T cell^7,8,11^. We found that the CD103^−^, CD69^+^ population of CD4^+^ T cells expanded early after *H. polygyrus* infection (Fig. 4r). Surprisingly, this subset persisted when total cell numbers normalised 10 weeks post-infection and 4 weeks after deworming, leaving fewer CD69^+^ CD103^+^ cells in the skin of long-term infected and dewormed mice (Fig. 4q, s, t). CCR8 have recently been suggested to define a CD4^+^ T_RM_ cell population in human skin^24^, but CCR8 was also reduced on skin CD4^+^ cells late after *H. polygyrus* infection or deworming (Supplementary Fig. 4m). Moreover, we did not find changes in CD11a and KLRG-1 (Supplementary Fig. 4n, o), which are associated with long-lived CD4^+^ cells^25^. In summary, our data show that activated T_H_2 cells, lacking several markers associated with T_RM_ cells, accumulate and persist in the skin of mice infected with *H. polygyrus*.

### Skin CD4^+^ cells are *H. polygyrus*-specific and travel through the circulation

**Fig. 5:**
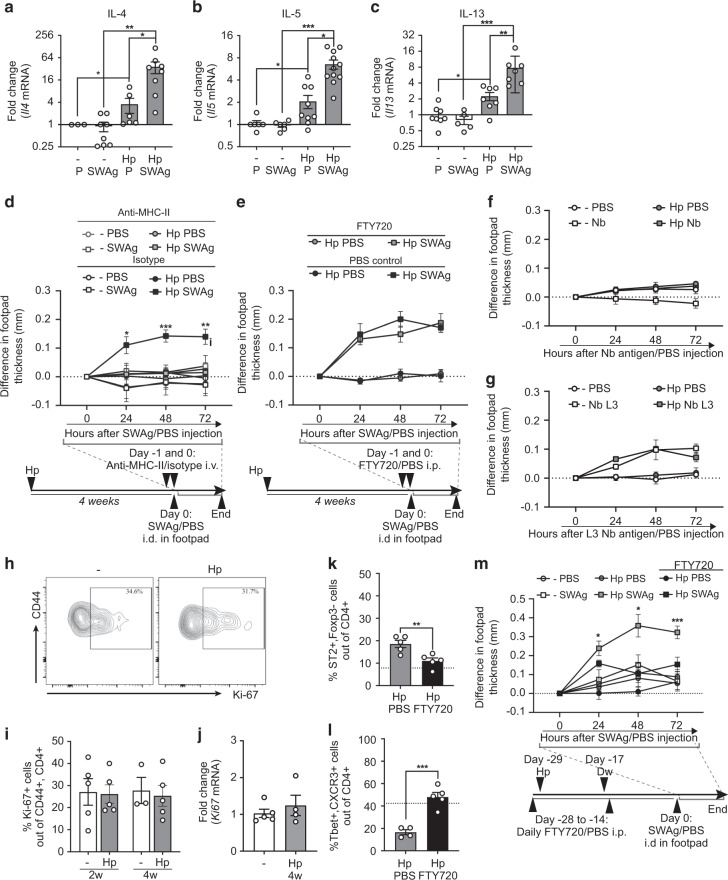
Skin CD4^+^ T cells display antigen-specific responses to *H. polygyrus*. Mice were infected with *H. polygyrus* (Hp) or not (−). **a**–**c** Soluble worm antigen (SWAg) from *H. polygyrus* (and PBS (P) in contralateral side as control) was injected in the back skin four weeks post infection. Fold change of mRNA expression of indicated cytokines analysed by qPCR. **d** Footpad swelling after SWAg injection in anti-MHC-II antibody or isotype treated mice and in **e** FYT720 or PBS treated mice as indicated. **f** Footpad swelling after injection of antigen from adult *N. brasiliensis* (Nb) or **g** stage L3 *N. brasiliensis* larvae (L3 Nb), 4 weeks post Hp infection. PBS was injected in the contralateral side as control. **h** Representative FACS plots of Ki-67^+^ cells gated on skin CD4^+^ , CD44^+^ T cells. **i** Frequency of Ki-67^+^ cells out of skin CD4^+^ , CD44^+^ T cells. **j** Fold change of *Ki67* mRNA expression analysed by qPCR. **k**–**m** Mice were injected daily with FTY720 or PBS ip 1 day prior to and 3 days after a 10 days infection with Hp. **k**, **l** Frequencies of T_H_2 (ST2^+^ , Foxp3^−^) and T_H_1 (Tbet^+^ , CXCR3^+^ ) cells out of CD4^+^ , CD44^+^ T cells (the dashed line show average levels in uninfected mice from previous experiments), 2 weeks after end of treatment. **m** Footpad swelling after SWAg injection in FYT720-treated and untreated mice as indicated. One out of at least two independent experiments with similar results are shown. Each dot represents the mean ± SEM of a group of mice (*n* = 5) in line graphs and an individual mouse in bar graphs where bars indicate the mean ± SEM. Statistical differences were analysed by Mann–Whitney U test (**a**–**c**) or unpaired *t*-tests (**d**, **k**–**m**) and depicted as *p < 0.05, **p < 0.01, ***p < 0.001. In **d** and **m** statistical comparisons are made to the Hp-infected, SWAg footpad injected but PBS ip injected group.

To investigate the reactivity of skin-localised CD4^+^ T cells in *H. polygyrus-*infected mice, we injected soluble worm antigen (SWAg) from *H. polygyrus* into the back skin of infected and uninfected mice and analysed cytokine mRNA expression by qPCR 24 h later. Baseline expression of *Il4*, *Il5* and *Il13* were approximately twofold higher in the skin of worm-infected mice compared to worm-free mice, indicating a low but continuous production of T_H_2 cytokines in the skin of worm-infected mice (Fig. 5a–c). Further, transcripts of these cytokines were clearly induced by SWAg injection in *H. polygyrus-*infected mice whereas no induction was seen in uninfected controls (Fig. 5a–c). We did not detect any changes in T_REG_ or T_H_1-associated cytokines (Supplementary Fig. 5a–d). To assess direct recall in the skin we measured footpad swelling elicited by SWAg. Indeed, worm-infected animals displayed a swelling reaction upon SWAg administration in the footpad skin, which was absent in uninfected mice (Fig. 5d). This reaction was MHC-II-dependent (Fig. 5d) and hence mediated by CD4^+^ T cells. We then depleted circulating lymphocytes using FTY720^6^ (Supplementary Fig. 5e) prior to the SWAg footpad injection to assess if SWAg-reacting lymphocytes were tissue-resident. FTY720 treatment did not affect SWAg-induced swelling in worm-infected mice (Fig. 5e), indicating that persisting local skin CD4^+^ T cells mediated the reaction. Many intestinal helminths infect by penetrating the skin, including the murine hookworm *Nippostrongylus brasiliensis*^26^*. H. polygyrus* infection has previously been shown to protect against subsequent challenges with *N. brasiliensis* at the skin-penetration stage^26^. We next injected antigen from *N. brasiliensis* in the footpad and measured footpad swelling, but no reaction was observed in *H. polygyrus*-infected mice (Fig. 5f, g), supporting antigen specificity of the accumulated CD4^+^ T cells. Incoherence with the CD4^+^ T specificity and lack of changes in ILC populations, no swelling reaction was induced by injection of the alarmin-inducing enzyme papain^27^ in the footpad (Supplementary Fig. 5f). In summary, CD4^+^ cells that accumulate in the skin of infected mice display *H. polygyrus*-specific reactivity and respond to worm antigen in an MHC-II-dependent manner by producing T_H_2-associated cytokines.

To further address the process of CD4^+^ T cells accumulation in skin, we measured the proliferation marker Ki-67 in skin CD4^+^ T cells at various time points and excluded that accumulation was a result of in situ proliferation (Fig. 5h–j, Supplementary Fig. 5g). Next, we again employed FTY720, this time prior to and throughout the worm infection to block cell migration from the intestine to skin, yet allowing potential worm derived molecules to circulate. The mice were dewormed 10 days after infection and FTY720 treatment ended 3 days after deworming, followed by a 2-week resting period allowing cells to equilibrate in the body. T_H_2 cells expanded in the skin of *H. polygyrus-*infected mice receiving PBS injections, whereas T_H_2 and T_H_1 cells remained at similar levels as those of worm-free controls in mice receiving FTY720 throughout the infection (Fig. 5k, l, average frequencies in worm-free mice are indicated by the dashed lines). The worm-induced eosinophil accumulation was not affected by the FTY720 treatment (Supplementary Fig. 5h). After the described FTY720 regimen, SWAg was injected into the skin of the footpad (Fig. 5m). SWAg induced a potent skin reaction in worm-infected mice, which was lacking in mice treated with FTY720 during the infection (Fig. 5m). Together, this indicates that *H. polygyrus*-specific CD4^+^ T cell accumulation in the skin is a result of cell migration from the intestine to skin rather than dissemination of soluble factors or in situ proliferation.

### *H. polygyrus*-specific footpad swelling and impaired IFN-*γ* responses to mycobacterial antigens persist after deworming

**Fig. 6:**
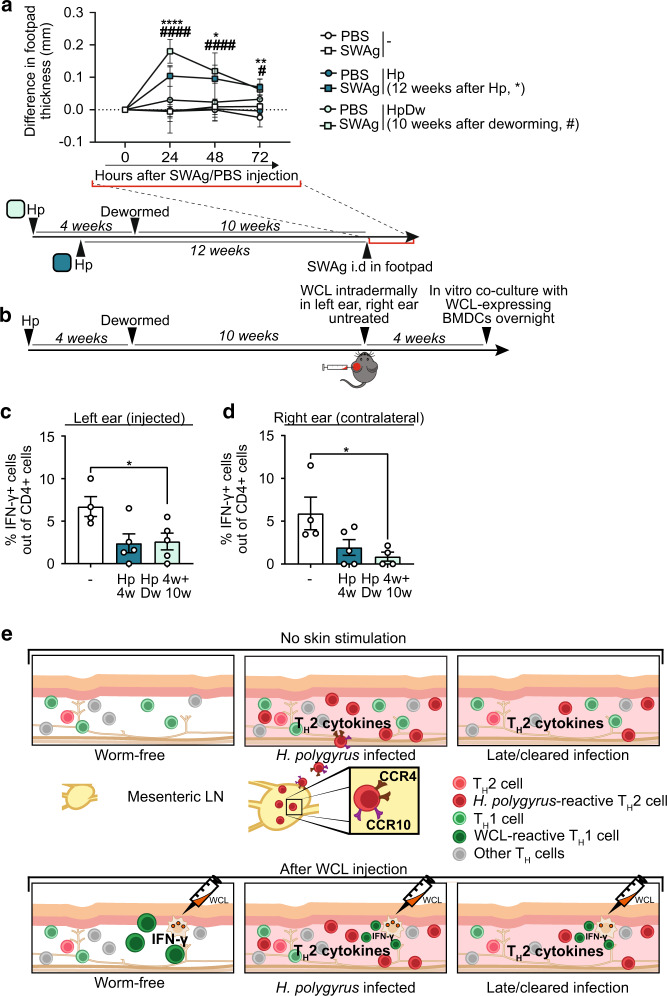
*H. polygyrus-*specific footpad swelling and muted skin IFNγ responses to mycobacterial antigens persist after removal of worms. Mice were infected with *H. polygyrus* (Hp) or not (-) and some dewormed (Dw) 4 weeks later. **a** Footpad swelling after soluble worm antigen (SWAg) from *H. polygyrus* injection in the footpad 12 weeks post infection. or 10 weeks after deworming. **b** shows the setup for **c** and **d** Mice were dewormed 4 weeks after Hp infection and 10 weeks later whole cell lysate (WCL) from *M. tuberculosis* was injected in the left ear and the right ear was left untreated. Four weeks later, ears were collected, and ear cells co-cultured with BMDCs expressing WCL overnight and analysed by flow cytometry. **c**–**d** Frequency of IFN-*γ*^+^ cells out of CD4^+^ T cells. **a**–**d** One out of at least two independent experiments with similar results are shown. Each dot represents the mean ± SEM of a group of mice (*n* ≥ 4 per group) in line graphs or an individual mouse in bar graphs, where bars indicate mean ± SEM. Statistical differences were analysed by unpaired *t*-tests (**a**, **c**) or Mann–Whitney U test (**d**) and depicted as *p < 0.05, **p < 0.01, ****p < 0.0001 or in a as above for 12 weeks Hp infected mice and as ^#^*p* < 0.05, ^####^*p* < 0.0001 for mice dewormed 10 weeks prior to SWAg injection. **e** shows a graphical summary of a suggested model for our findings. Upper panel: Intestinal *H. polygyrus* infection primes worm-specific CD4^+^ T cells into CCR4^−^ and CCR10-expressing T_H_2 cells in the mesLNs. Primed cells travel through the circulation to the skin where they remain after the infection is cleared and total numbers have normalised, persistently transforming the skin CD4^+^ T cell pool. Lower panel: Intradermal WCL injection causes mycobacteria-reactive T_H_1 cells to accumulate in the skin. The increased levels of T_H_2 cytokines present in the skin of worm infected or worm-cleared mice dampen the functionality of T_H_1 cells, muting their capacity to produce IFN-*γ* in response to restimulation with mycobacterial products.

Our previous studies showed that deworming can in 3 weeks restore immune reactivity of the skin-draining LNs dampened by *H. polygyrus* infection^6^. To test if the altered composition of skin T cells seen weeks after clearance of worms would affect subsequent responses, we injected SWAg in the footpad 12 weeks after *H. polygyrus* infection or 10 weeks after deworming (Fig. 6a). Mice that had been infected with *H. polygyrus* retained a noticeable skin reaction upon SWAg injection at both timepoints (Fig. 6a). Furthermore, in mice previously infected but dewormed 10 weeks prior to WCL injection in the skin, IFN-*γ* recall responses were still impaired at both WCL-injected and untouched skin sites (Fig. 6b–d). This implies that *H. polygyrus* infection in the gut causes durable changes in the composition of skin T cells and persistently alters the capacity to form skin responses to an unrelated antigen (Fig. 6e).

## Discussion

Intestinal worm infection impacts immune responses to other pathogens, vaccines, autoantigens, allergens and contact sensitisers^2,6,13,28^. These effects have been believed to be due to expansion of T_REG_^2,28,29^ and/or T_H_2 cells^21,30^, atrophy of peripheral LNs^6,31^ or changes in the intestinal microbiota or its metabolome^32–34^. Yet, the full spectrum of helminth-induced effects on the immune system is not elucidated. Our findings add to the growing body of literature describing how worms can form our immune system and cause extraintestinal immunosuppression^2,6,13,21,28,34^ and propose new ways by which gut-restricted worms impact the onset of skin immune responses. We show that infection with the intestinal nematode *H. polygyrus* promote homing to and long-term residence of *H. polygyrus*-specific T_H_2 cells producing IL-4 and IL-13 in the skin. We propose that this gut infection-induced remodelling of the skin T cell landscape led to dampened skin recall responses to unrelated antigens, both locally and distally to the injection site. Different to the reversible effect removal of intestinal *H. polygyrus* infection had on skin-draining lymph nodes^6^, the impaired skin T cell response and T_H_2 skewing persisted long after worms had been cleared from the host. IFN-*γ* responses in other organs have previously been shown to be muted in helminth-infected humans and animals^4,13,35^. For example, intestinal helminth-induced suppression of immunity towards *M. tuberculosis* has been shown to be mediated via the common IL-4/IL-13 receptor (IL-4R*α*) pathway in the lung^30^, and a similar mechanism is a possibility also in our model.

Several mechanisms have been suggested to drive cell trafficking into tissues distinct from the infection site. Studies have proposed the dissemination of pathogen products^36^, re-programming of lymphocyte homing profile by a new tissue or LN microenvironment^37–40^ and up-regulation of chemokine receptors on cells in LNs draining the infection site that target them for homing to sites distal to the infection^41^. *H. polygyrus* driven accumulation of CD4^+^ T cells in the skin was found not to be a consequence of bacterial translocation into the blood, excretion of worm antigens into the environment, or dissemination of soluble worm molecules. Instead, *H. polygyrus* infection triggered expression of skin-homing CCR4 and CCR10 on CD4^+^ T cells in mesLNs and blood, indicating a more direct link between gut and skin. Furthermore, worm-specific T_H_2 cell accumulation was lost following FTY720 treatment during infection. Long-term DSS treatment, known to induce a T cell-driven inflammatory response in the intestine^18^, did not cause CD4^+^ T cell deposition in the skin, and neither was skin-homing receptors induced by the treatment. Our data indicate that the higher number of T_H_2 cells lodged in the skin of *H. polygyrus*-infected animals is a result of induction of skin-homing chemokine receptor expression on CD4^+^ T cells in the mesLN causing intestine-to-skin cell trafficking via the circulation.

The functional advantage of the observed recruitment of CD4^+^ T cells into the skin is not clear. A number of worm species infect through the skin^42^, thus, skin-homing T_H_2 cells could provide direct protection against repeated exposure to larval forms of the same skin-infecting nematode or cross-protection against other worms. Indeed, *H. polygyrus* have been shown to protect against challenge by the *N. brasiliensis*^26,43^ and cross-protection between helminth species have also been seen in other models^44,45^. We did not observe cross-reactivity to *N. brasiliensis* antigens in our model as measured by footpad swelling. Prior studies point to innate type 2 responses rather than CD4^+^ T cells as mediators of such cross-reactivity^26,44,45^. In our model, we did not find any differences in ILC populations or sensitivity to papain, which might explain the lack of cross-reactivity. The skin responses we detected were MHC-II-dependent with evident IL-4 and IL-13 production induced after restimulation, revealing a strong *H. polygyrus*-specific T_H_2 component among the CD4^+^ T cells accumulating in the skin. Importantly, T_H_2 cells persisted in the skin even after the expulsion of *H. polygyrus* from the gut and similarly, skin recall responses to a secondary challenge with a mycobacterial antigen preparation were muted long after worm expulsion. The changed composition of skin CD4^+^ T cells and compromised ability of worm-infected mice to mount skin reactions to subsequent antigen challenge, can have consequences for immune responses to pathogens and vaccines. Intriguingly, our results propose a novel explanation to the geographically mutually exclusive high prevalence of inflammatory skin disorders and the responsiveness to the intradermally administered tuberculosis vaccine BCG, observed around the world^3,4,35^.

## Methods

### Mice and ethics

Three to 4 weeks old C57BL/6NRj mice were obtained from Janvier Labs (France), Taconic Biosciences (Denmark) or Karolinska Institutet and acclimatised for at least 1 week before the start of the experiment. Mice were housed and handled under SPF conditions at the BSL-2 facility at Comparative Medicine Biomedicum (KM-B) according to Swedish national regulations for laboratory animal work with free access to food and water, cage enrichment (nesting material, gnaw stick) and 12 h light and dark cycles. Mice were anaesthetised with isoflurane during skin injections. Female mice were used in most experiments. The experimental group size was determined based on previous experience of variation (often one cage of five animals, as provided to us) and mice/cages allocated randomly into groups. Experiments were not blinded at any stage. Experiments were granted by the regional ethical board (Stockholms djurförsöksetiska nämnd) under permit numbers 6738/19, N89/15 and N171/14 with the amendment N131/16 and exemption from L150 Dnr 5.2.18-7344/14 approved by the Swedish Board of Agriculture.

### Skin collection

The back skin of euthanized mice was shaved and Veet® hair removal cream was applied onto the shaved area. The cream was removed after 3–5 min, and the treated area was washed with PBS. Back skin was collected with biopsy punches. Ears were removed with scissors without prior shaving or treatment.

### Infections and in vivo recall responses

Mice were infected with 300 L3 stage larvae of *H. polygyrus* (except for in Fig. 1 where 200 larvae were used) per oral gavage at 4 to 5 weeks of age. At the end of each experiment, adult worms were collected as previously described^6^ for enumeration and antigen preparations (see below). Deworming was done with 2 mg Fyrantel® paste (0.8 mg pyrantel pamoate) per mouse for 3 constitutive days and successful treatment was confirmed by lack of eggs in the faeces. Ears were injected intradermally with 5 µg WCL (*M. tuberculosis* strain H37Rv, NR-14822, BEI Resourses, NIAID, NIH) in 5 µl PBS. For recall responses, worm antigen was injected in footpads, and footpad swelling was measured with a digital caliper or in shaved and Veet®-treated back skin for cytokine assessment by qPCR. Worm antigens were administered in 20 µl to back skin or footpad at 50 µg (SWAg or adult *N. brasiliensis*) or 25 µg (L3 *N. brasiliensis*) and papain (Merck) at 25 µg. Soluble worm antigen (SWAg) from *H. polygyrus* and antigens from *N. brasiliensis* was prepared as previously described^13,46^ and stored at −80 °C.

### Dextran sodium sulphate model

Dextran sodium sulphate (DSS, TdB Consultancy AB) was provided at 2% w/v *ad libitum* in the drinking water for 1 or 2 periods of 7 days each with a recovery period of 14 days in between for the latter. Mice were sacrificed 7 days following the first or second DSS period.

### Antibody and FTY720 treatments

Anti-MHC-II (400 μg/injection, produced inhouse by L. Boon) was injected intravenously and Fingolimod/FTY720 (3 mg/kg, Sigma Aldrich) intraperitoneally the day before and just before SWAg footpad injection. In some experiments, FTY720 was injected from 1 day prior to *H. polygyrus* infection and then daily for 14 days.

### CD4^+^ T cell bead isolation

MesLNs were collected and CD4^+^ T cells isolated using the Dynabeads™ Untouched™ Mouse CD4 Cells Kit (Thermo Fisher) according to the manufacturer’s instructions.

### Gene expression by qPCR

A total of 4 mm back skin biopsies were collected into QIAzol^TM^ lysis reagent (Qiagen) and chopped into smaller pieces. Skin samples were homogenised in a TissueLyser LT (Qiagen, 50 Hz, 2 times 4 min) using 5 mm stainless steel beads (Qiagen). RNA was extracted by the chloroform/isopropanol method and converted to cDNA as previously described^13^. qPCR was performed using TaqMan technology (Applied Biosystems) with primers and probe kits from Life technologies (Supplementary Table 1) and run on a CFX384 qPCR machine (BioRad). Target gene expression data were normalised to the expression of *β*-actin for individual samples and fold change over control samples calculated by using the 2^−ΔΔCt^ method.

### Flow cytometry

Back skin and ears were cut into smaller pieces and incubated in collagenase 3 (3 mg/ml, Worthington), DNAse I (5 µg/ml, Roche) and 10% FBS (Sigma) in RPMI-1640 at 37 °C, 5% CO_2_ for 90 min. Samples were then grinded in BD Medimachine for 4 min, filtered, and washed. Blood was collected into EDTA-containing tubes (Sarstedt), red blood cells (RBCs) lysed and cells washed. Spleens were homogenised through 70 µm strainers with a syringe plunger, RBCs lysed, and cells. Single-cell suspensions were stained with LIVE/DEAD™ Fixable Yellow Dead Cell Stain according to manufacturers’ instructions (Invitrogen), washed, and incubated with combinations of antibodies to CD45 (30-F11), CD4 (RM4-5 or GK1.5), CD8 (53–6.7), KLRG-1 (2F1), CD44 (IM7), Siglec-F (E50-2440) and I-A/I-E (2G9) from BD, TCRβ (H57-597), PD-1 (29 F.1A12), ST2 (DIH9), CCR8 (SA214G2), CCR4 (2G12), CD69 (H1.2F3), CD103 (2E7), *γ*δ TCR (GL3), CD11c (N418), Ly6G (RB6-8C5) and CD183 (CXCR3-173) from Biolegend, CCR10 (248918) from R&D, CD11a (M17/4) from eBioscience, FcεRI (MAR-1), c-Kit (CD117, 2B8), CD11b (M1/70) and F4/80 (BM8) from Invitrogen and Mouse BD Fc Block™, for 30 min at 4 °C. For transcription factors, cells were fixed and permeabilized with the Foxp3 staining kit (eBioscience) or IntraPrep Permeabilizaton Reagent (Beckman Coulter) and then stained with combinations of Foxp3 (FJK-16s), Ki-67 (SolA15) from Invitrogen and ROR*γ*t (Q31-378) and Tbet (4B10) from BD. To assess cytokine production, PMA (50 ng/ml), ionomycin (5 μM) and Brefeldin A (5 μg/ml, all Sigma) were added to the cells for 3 h. For WCL restimulation, bone marrow-derived DCs (BMDCs) were isolated as previously described^47^, incubated with WCL (10 µg/ml) overnight, and ear cells added the following day. The co-culture was incubated overnight, and Brefeldin A added for the last 3 h. Stimulated cells were fixed and permeabilized using the Foxp3 staining kit (eBioscience) or IntraPrep Permeabilizaton Reagent (Beckman Coulter) and stained with combinations of IL-13 (eBio13A) from Invitrogen and IL-4 (11B11) and IFN-*γ* (XMG1.2) from BD. Cells were analysed on LSR II flow cytometer (BD). Absolute numbers of cells were calculated by using CountBright™ Absolute Counting Beads (Invitrogen) or in haemocytometer and data analysed in FlowJo V10 (BD).

### Soluble CD14 (sCD14) ELISA

sCD14 levels were determined in mouse serum by ELISA (Biometric Gmbh) according to the manufacturer’s instructions.

### Statistics and data presentation

Outliers plausibly appearing from technical errors or unknown reasons were excluded prior to analysis by the ROUT test as recommended by GraphPad Prism to eliminate subjectivity when evaluating the validity of data as determined a priori, as confounders were not explicitly controlled. For statistical comparison between two groups, unpaired student’s *t* test was performed when data followed normal distribution and Mann–Whitney U when distributions did not. One-way ANOVA with Tukey post-test was used for multiple comparisons. GraphPad Prism version 8 or 9 were used.

## Supplementary information

Supplementary information
