# Supplementary material for: Intestinal helminth infection transforms the CD4+ T cell composition of the skin
Source: Mucosal Immunol. 2021 Dec 20;15(2):257–67. doi: 10.1038/s41385-021-00473-9 (PMC8866128; doi:10.1038/s41385-021-00473-9)
Supplement: Supplementary file 1 — Supplementary information [file 41385_2021_473_MOESM1_ESM.pdf]

Supplementary Figure 1

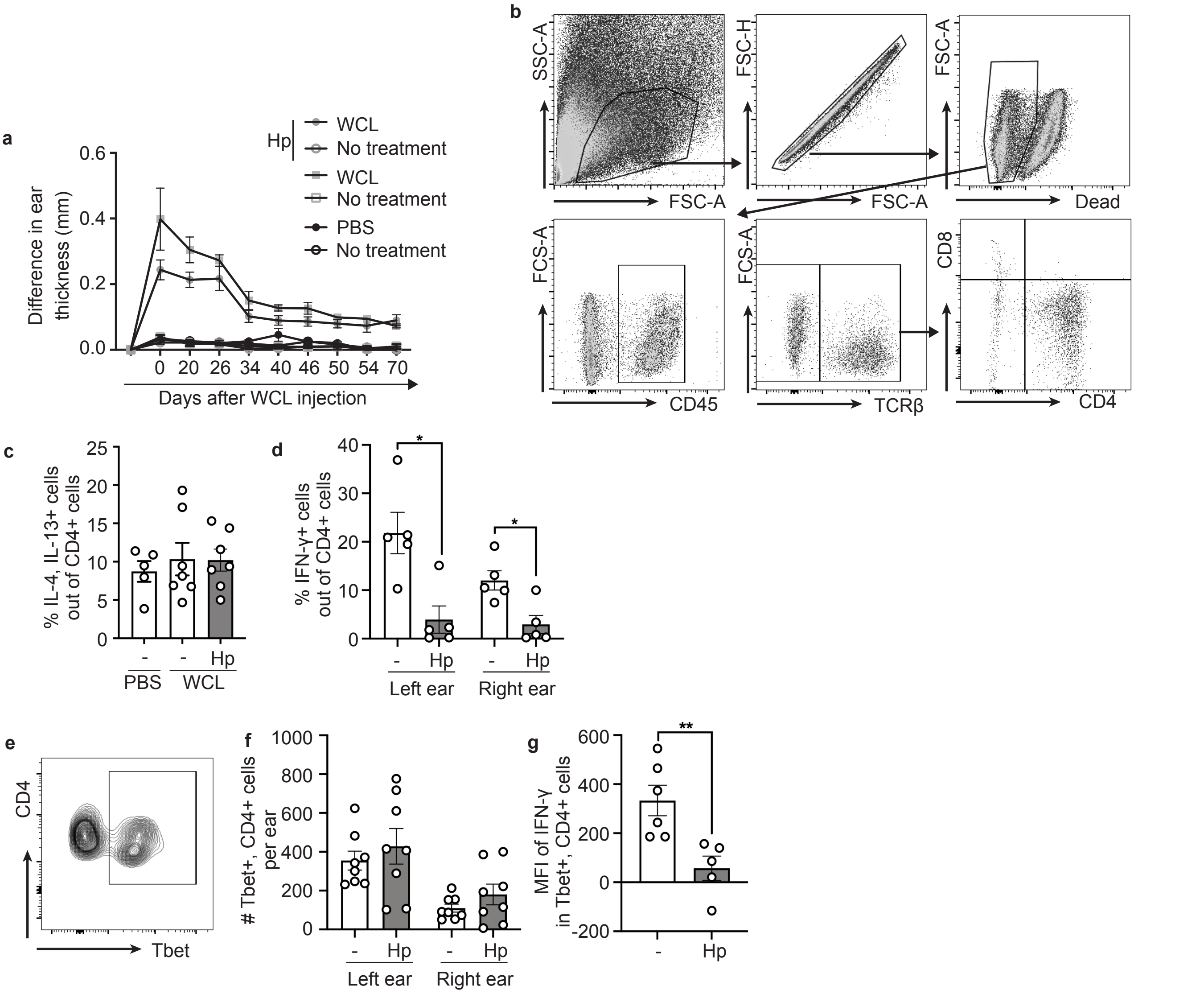

Supplementary Figure 1: Complement to Figure 1. Mice were infected with *H. polygyrus* (Hp) or not (-) and ears injected with whole cell lysate (WCL) according to Fig. 1a. **a** Ear thickness measured after WCL injection in ears. **b** Representative gating strategy used for CD4+ T cells in ears. **c** Frequencies of IL-4+, IL-13+, CD4+ T cells in left ears after PBS or WCL injection. **d** Frequencies of IFN-γ+ CD4+ T cells after PMA/ionomycin restimulation in the presence of Brefeldin A. **e** Representative FACS plot of Tbet+ CD4+ T cells. **f** Absolute number of Tbet+, CD4+ T cells per ear. **g** Median flourescent intencity (MFI) of IFN-γ+ in left ear CD4+ T cells. Each dot represents the mean ± SEM of a group of mice in line graphs and an individual mouse in bar graphs where bars indicate the mean ± SEM. Statistical differences were analysed by Mann-Whitney U test (**c**) or unpaired t-tests (**e, f**) and are depicted as $^*p < 0.05$, $^{**}p < 0.01$.

Supplementary Figure 2

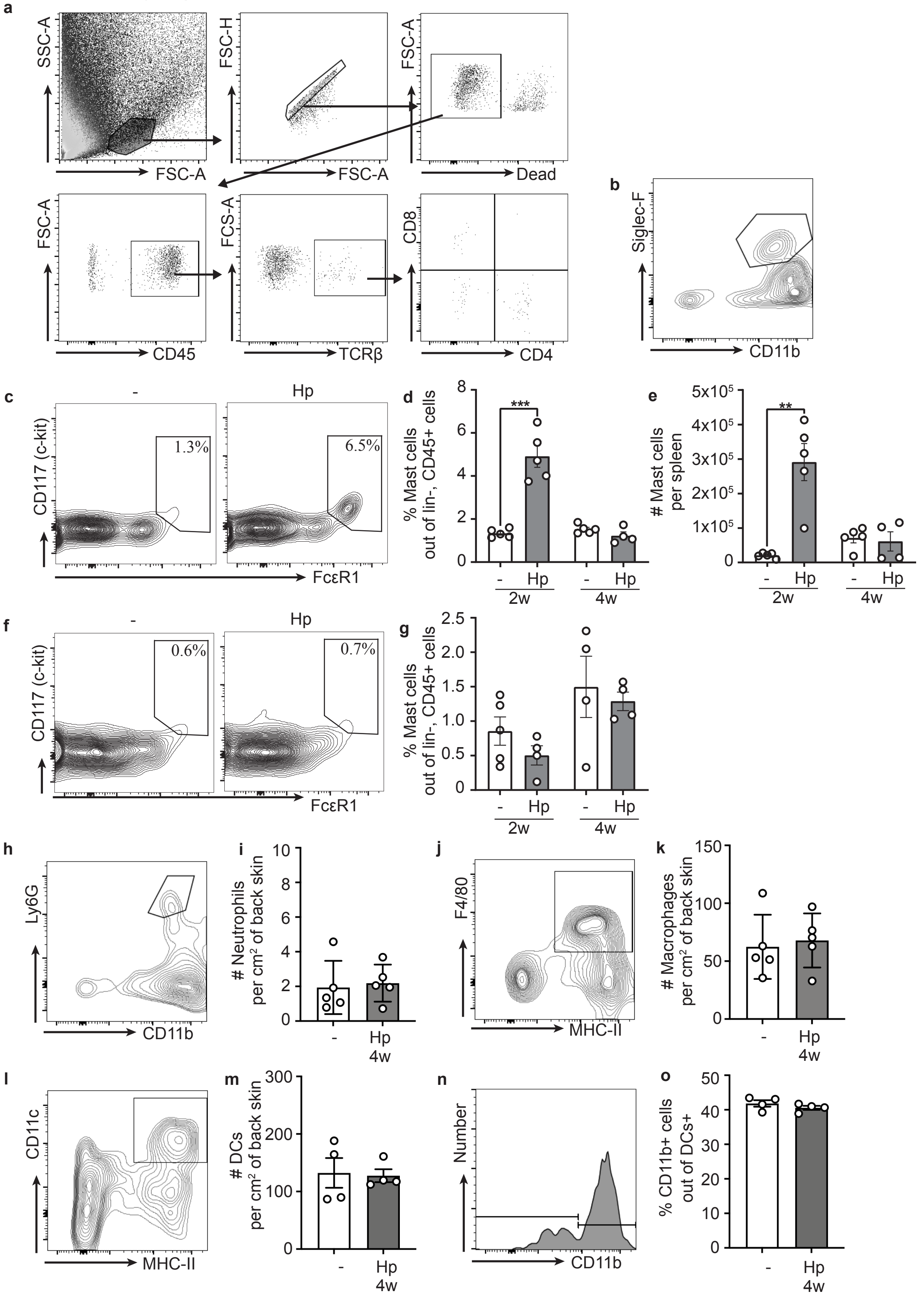

Supplementary Figure 2 (continuation)

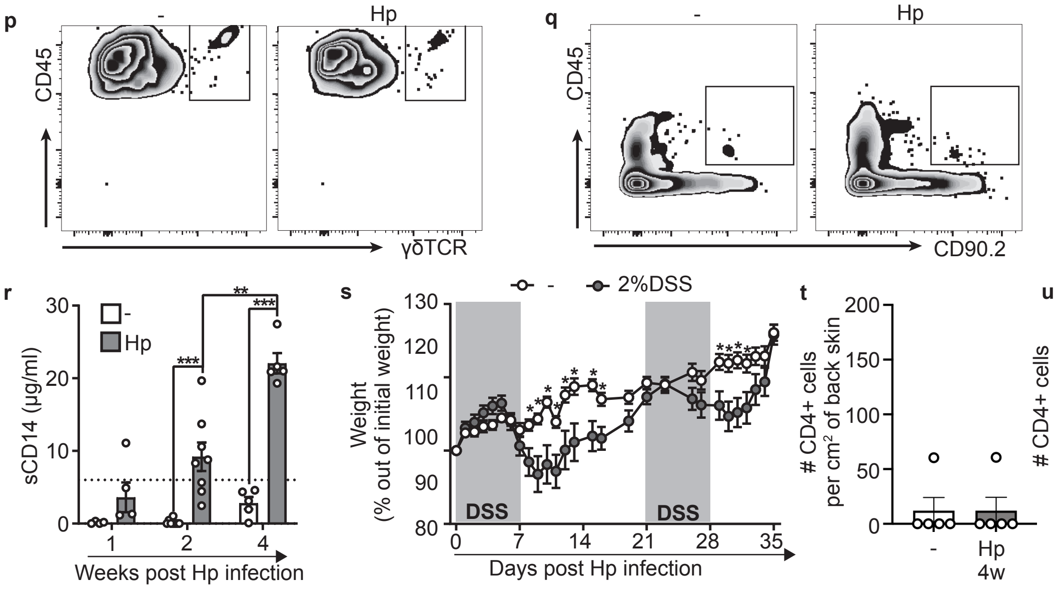

Supplementary Figure 2: Complement to Figure 2. a Representative gating strategy used for CD4+ T cells in back skin. **b-r** Mice were infected with *H. polygyrus* (Hp) or not (-). **b** Representative FACS plot of eosinophils (CD11b+, Siglec-F+, gated on CD45+). **c-e** Representative FACS plots, frequencies and absolute numbers of mast cells (c-Kit+, FcεRI+, gated on linage- (lin-), CD45+ cells) in spleen. **f, g** Representative FACS plot and frequencies of mast cells in back skin. **h, i** Representative FACS plot and absolute number of neutrophils (CD11b+, Ly6G^{HIGH}, gated on CD45+) in back skin. **j, k** Representative FACS plot and absolute number of macrophages (F4/80+, MHC-II, gated on CD11b+, CD45+) in back skin. **l, m** Representative FACS plot and absolute number of DCs (CD11c, MHC-II, gated on CD45+) in back skin. **n, o** Representative FACS plot and frequncies of CD11b+ DCs in back skin. **p** Representative FACS plot of γδTCR+ T cells in back skin. **q** Representative FACS plot of innate lymphoid cells (ILCs, CD45+, CD90.2+, gated on lineage negative cells) in back skin. **r** Soluble (s) CD14 levels measured by ELISA in serum. The dotted line represents the normal cut-off value for an uninfected mouse. **s** Weight of mice treated with 2% dextran sodium sulfate (DSS) in the drinking water and indicated timepoints after start of treatment. Periods of treatment are shown in gray. **t, u** p25-TCR Tg mice were infected with *H. polygyrus* (Hp) or not (-). Absolute numbers of CD4+ T cells in back skin and mesenteric lymph nodes of p25-TCR Tg mice. **c-r, t, u** One out of at least two independent experiments with similar results are shown. In **s**, the experiment has been performed once but with 10 mice (divided in 2 cages) per group. Each dot represents the mean ± SEM of a group of mice in line graphs and an individual mouse in bar graphs where bars indicate the mean ± SEM. Statistical differences were analysed by unpaired t-tests (**d, e, r, s, u**) and depicted as *p < 0.05, **p < 0.01, ***p < 0.001.

Supplementary Figure 3

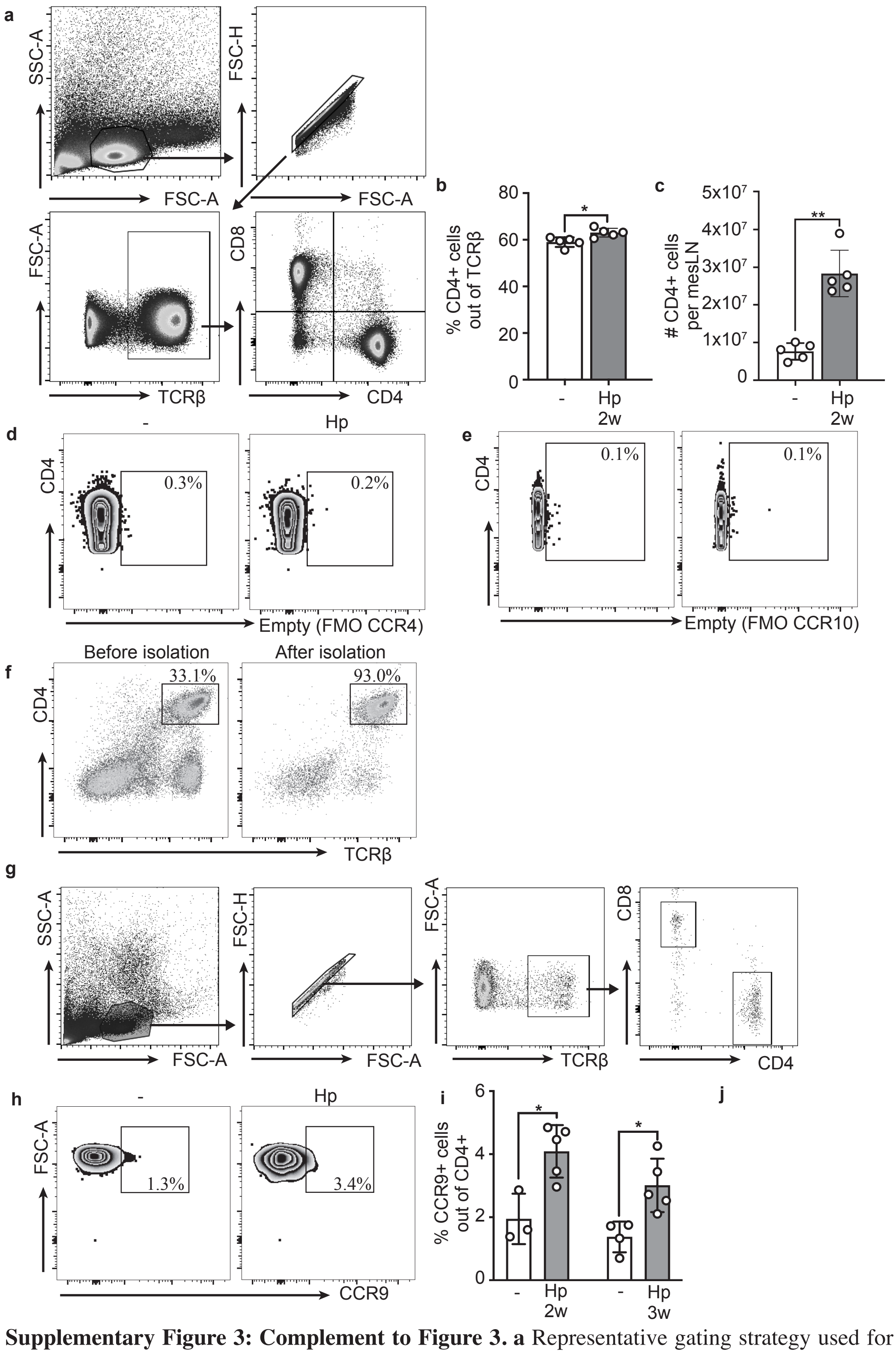

Supplementary Figure 3: Complement to Figure 3. a Representative gating strategy used for CD4+ T cells in mesenteric lymph nodes (mesLNs). **b-i** Mice were infected with *H. polygyrus* (Hp) or not (-). **b, c** Frequency and absolute numbers of CD4+ T cells in mesLNs. **d, e** FMO controls for CCR4 and CCR10 staining, respectively. **f** FACS plots showing purity of CD4+ T cells after bead isolation for the qPCR analysis in Fig. 3g and 3h. **g** Representative gating strategy used for CD4+ T cells in blood. **h** Representative FACS plot of CCR9+ staining in blood. **i** Frequencies of CCR9+ cells out of blood CD4+ T cells. One out of at least two independent experiments with similar results are shown. Each dot represents a mouse (n≥3 per group) and bars indicate the mean ± SEM. Statistical differences were analysed by unpaired t-tests and depicted as *$p < 0.05$, **$p < 0.01$.

Supplementary Figure 4

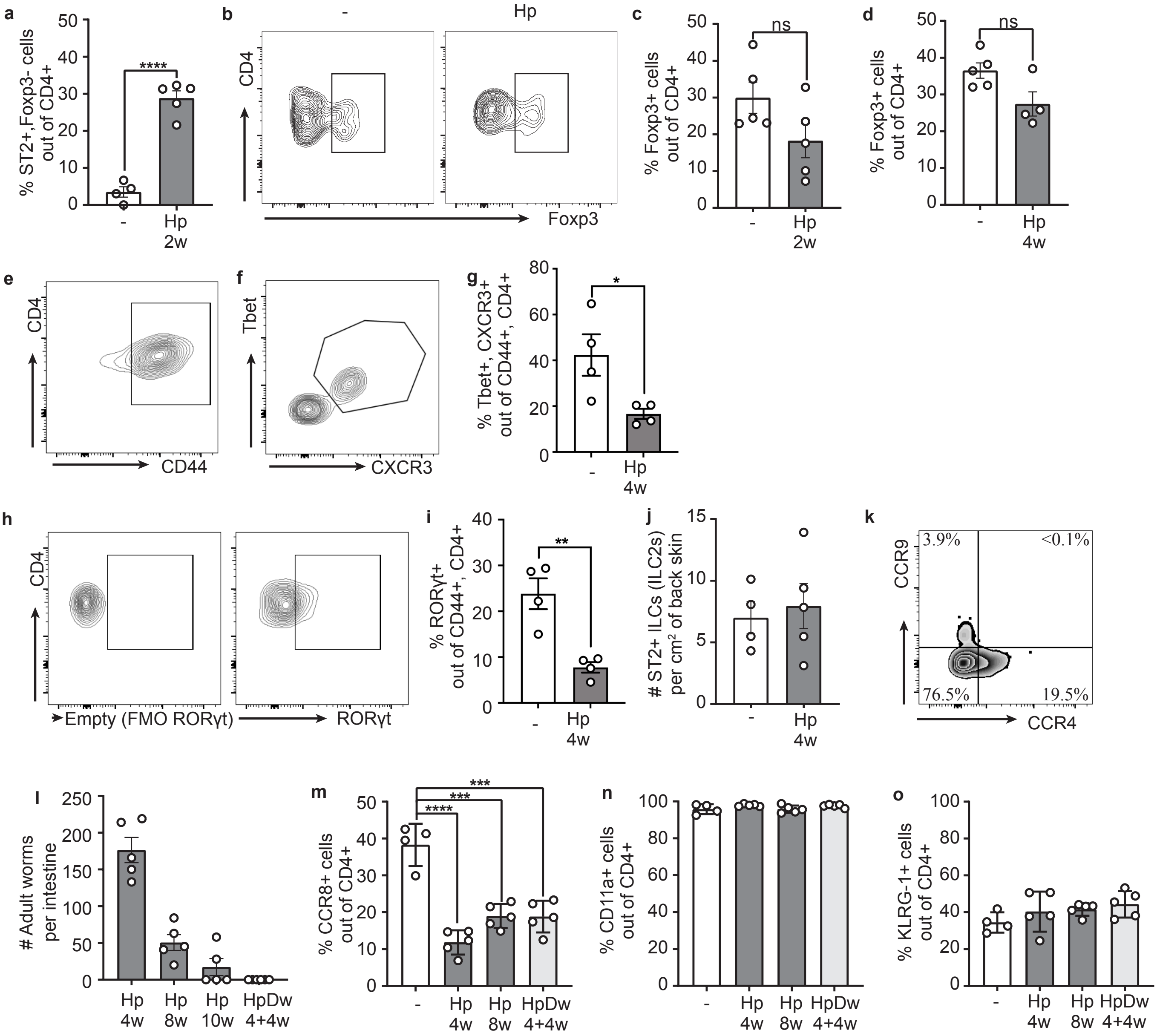

Supplementary Figure 4: Complement to Figure 4. Mice were infected with *H. polygyrus* (Hp) or not (-) and some dewormed (Dw) 4 weeks later. **a-n** Flow cytometry on back skin. **a** Frequency of ST2+, Foxp3- (T_H2) cells out of CD4+ T cells. **b-d** Representative FACS plots and frequencies of Foxp3 staining in CD4+ T cells. **e** Representative gating strategy for the CD44+ population out of CD4+ T cells used for Fig. 4c. **f, g** Representative FACS plots and frequencies of T_H1 cells (Tbet+, CXCR1+) in CD44+, CD4+ T cells. **h, i** Representative FACS plots and frequencies of T_H17 (RORγt+) in CD44+, CD4+ T cells. (Representative FACS plots and frequencies of T_H2 cells (ST2+, Foxp3-) and T_{REGs} on CD44+ cells is not shown since its close to identical to the gating in all CD4+ cells (Fig. 4 and Supplementary Fig. 4b, respectively) as almost all CD4+ cells express CD44 in the skin. **j** Absolute numbers of ST2+ ILCs. **k** Representative FACS plot of CCR4 and CCR9 expression in mesLN ST2+, CD4+ T cells. **l** Adult worms counted in small intestines at indicated time points after infection with 300 L3 larvae. **m-o** Frequencies of CCR8+, CD11a+, and KLRG1+ cells out of CD4+ T cells. One out of at least two independent experiments with similar results are shown. Each dot represents an individual mouse (n≥4 per group) and bars indicate the mean ± SEM. Statistical differences were analysed by unpaired t-tests and are depicted as ns = non-significant, *$p < 0.05$, **$p < 0.01$, ***$p < 0.001$, ****$p < 0.0001$.

Supplementary Figure 5

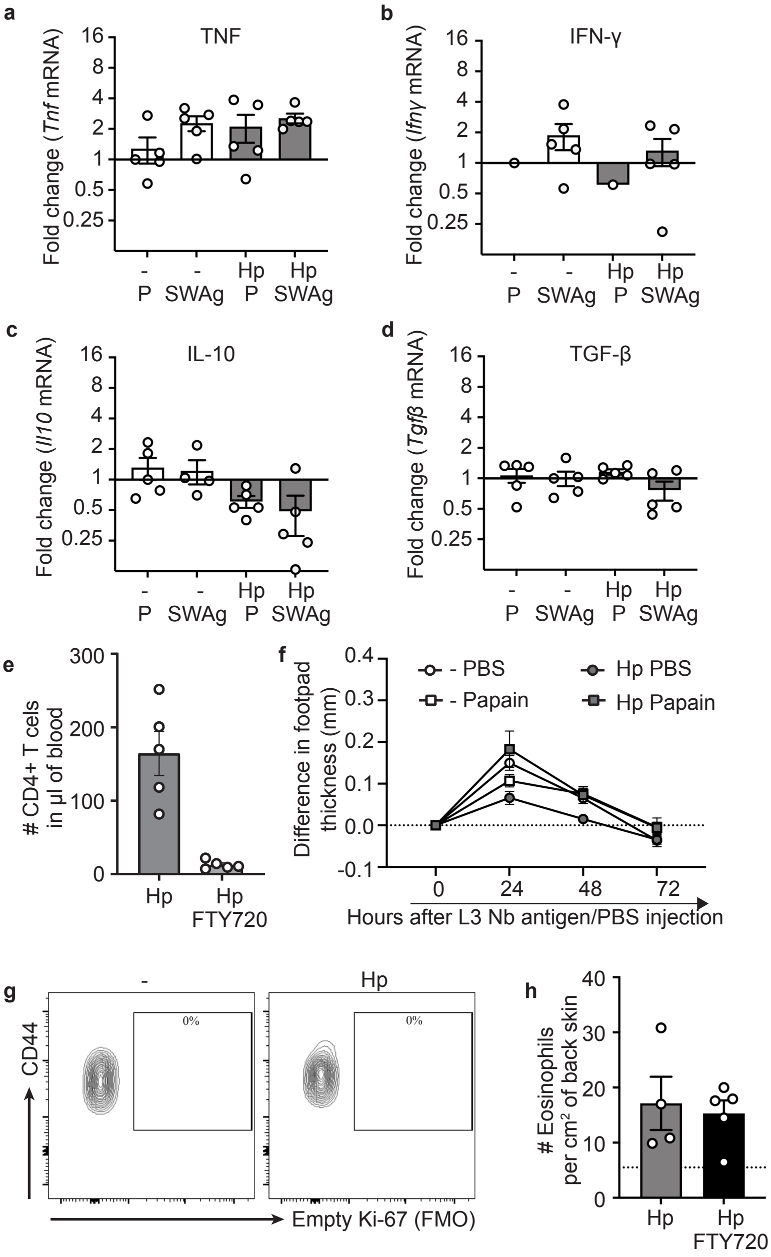

Supplementary Figure 5: Complement to Figure 5. Mice were infected with *H. polygyrus* (Hp) or not (-). **a-d** Soluble worm antigen (SWAg) from *H. polygyrus* (and PBS (P) in contralateral side as control) was injected in the back skin 4 weeks post infection. Fold change of mRNA expression of indicated cytokines analysed by qPCR. Five mice per group were always used, and when fewer dots are shown expression was under detection levels. **e** Number of CD4+ T cells in blood at the time of SWAg footpad injection (one day after first FTY720 treatment for Fig. 5e) counted by flow cytometry. **f** Footpad swelling after injection of papain 4 weeks post infection. PBS was injected in the contralateral side as control. **g** FMO controls for Ki-67 staining of back skin. **g** Absolute number of eosinophils (CD45+, CD11b+, Siglec-F+) in back skin (the dashed line show average levels in uninfected mice from previous experiments). One out of at least two independent experiments with similar results are shown. Each dot represents the mean ± SEM of a group of mice in line graphs and an individual mouse in bar graphs where bars indicate the mean ± SEM.

Supplementary Table 1: TaqMan gene expression assay numbers

**Gene**	**TaqMan Assay Number**
Ccr4	*Mm01963217_u1*
Ccr10	*Mm01292449_m1*
Ifng	*Mm01168134_m1*
Il4	*Mm00445259_m1*
Il5	*Mm00439646_m1*
Il10	*Mm01288386_m1*
Il13	*Mm00434204_m1*
Mki67	*Mm01278617_m1*
Tgfb1	*Mm01178820_m1*
Tnf	*Mm00443258_m1*
